# Different hydration methods for the prevention of contrast-induced nephropathy in patients with elective percutaneous coronary intervention: a retrospective study

**DOI:** 10.1186/s12872-023-03358-w

**Published:** 2023-06-24

**Authors:** Fei Chen, Jingchao Lu, Xiuchun Yang, Demin Liu, Qian Wang, Xue Geng, Bing Xiao, Jie Zhang, Fan Liu, Guoqiang Gu, Wei Cui

**Affiliations:** Department of Cardiology, the Second Hospital of Hebei Medical University and the Institute of Cardiocerebrovascular Disease of Hebei Province, Shijiazhuang, 050000 China

**Keywords:** Contrast-induced nephropathy, Coronary heart disease, Percutaneous coronary intervention, Hydration

## Abstract

**Background:**

Hydration is currently the main measure to prevent contrast-induced nephropathy (CIN). We aimed to compare the preventive effect of preprocedure and postprocedure hydration on CIN in patients with coronary heart disease undergoing elective percutaneous coronary intervention (PCI).

**Methods:**

A retrospective study included 198 cases of postprocedure hydration and 396 cases of preprocedure hydration using propensity score matching. The incidence of CIN 48 h after PCI and adverse events within 30 days after contrast media exposure were compared between the two groups. Logistic regression analysis was used to analyse the risk factors for CIN.

**Results:**

The incidence of CIN in the postprocedure hydration group was 3.54%, while that in the preprocedure hydration group was 4.8%. There was no significant difference between the two groups (*p* = 0.478). Multivariate logistic regression analysis showed that diabetes mellitus, baseline BNP and cystatin C levels, and contrast agent dosage were independent risk factors for CIN. There was no significant difference in the incidence of major adverse events between the two groups (3.03% vs. 2.02%, *p* = 0.830).

**Conclusions:**

Postprocedure hydration is equally effective compared to preoperative hydration in the prevention of CIN in patients with coronary heart disease undergoing elective PCI.

**Supplementary Information:**

The online version contains supplementary material available at 10.1186/s12872-023-03358-w.

## Introduction

Coronary angiography (CAG) is the commonly used diagnostic method for coronary heart disease (CHD), and percutaneous coronary intervention (PCI) is one of the most effective treatments for severe CHD. In recent years, with the increase in the number of PCIs and the improvement of PCI techniques, the treatment of complex coronary lesions and chronic total occlusion (CTO) is not a difficult problem. However, it is inevitable to use contrast media (CM) in percutaneous coronary intervention, especially for complicated coronary lesions and CTO, and more CM is needed. Contrast-induced nephropathy (CIN) was defined as the elevation of serum creatinine ≥ 0.5 mg/dl (44 μmol/l) or a more than 25% increase above baseline 48–72 h after exposure to CM [1]. CIN is a common cause of hospital-acquired acute renal insufficiency, which leads to increased mortality, hospitalization expenses and prolonged hospitalization time. Once CIN occurs, there is no specific treatment, so the goal is prevention. The single most important measure to reduce the incidence of CIN is hydration [1]. There are several specific hydration strategies, such as intravenous normal saline, sodium bicarbonate, the Guard System and haemodynamic guidance. Although researchers have not yet established an optimal strategy, experts generally suggest that hydration should be based on the intravenous administration of normal saline because no significant advantages have been demonstrated for other solutions [2, 3]. Guidelines and consensus suggest that 1 ml/kg/h (0.5 ml/kg/h if  LVEF ≤ 35%) saline should be maintained 4–12 h before PCI and sustained 6–24 h after PCI [4, 5]. Preprocedure hydration time is adequate for inpatients; however, it is limited for emergency PCI patients and outpatients. Furthermore, classic hydration is inconvenient for patients and inaccurate for CIN prevention. To our knowledge, we have not found any studies comparing the effect of standard hydration and postprocedure hydration on CIN prevention.

The purpose of this retrospective study was to compare the effect of preprocedure standard hydration versus postprocedure hydration on the prevention of CIN in patients with CHD undergoing elective PCI.

## Methods

### Study population

**Fig. 1:**
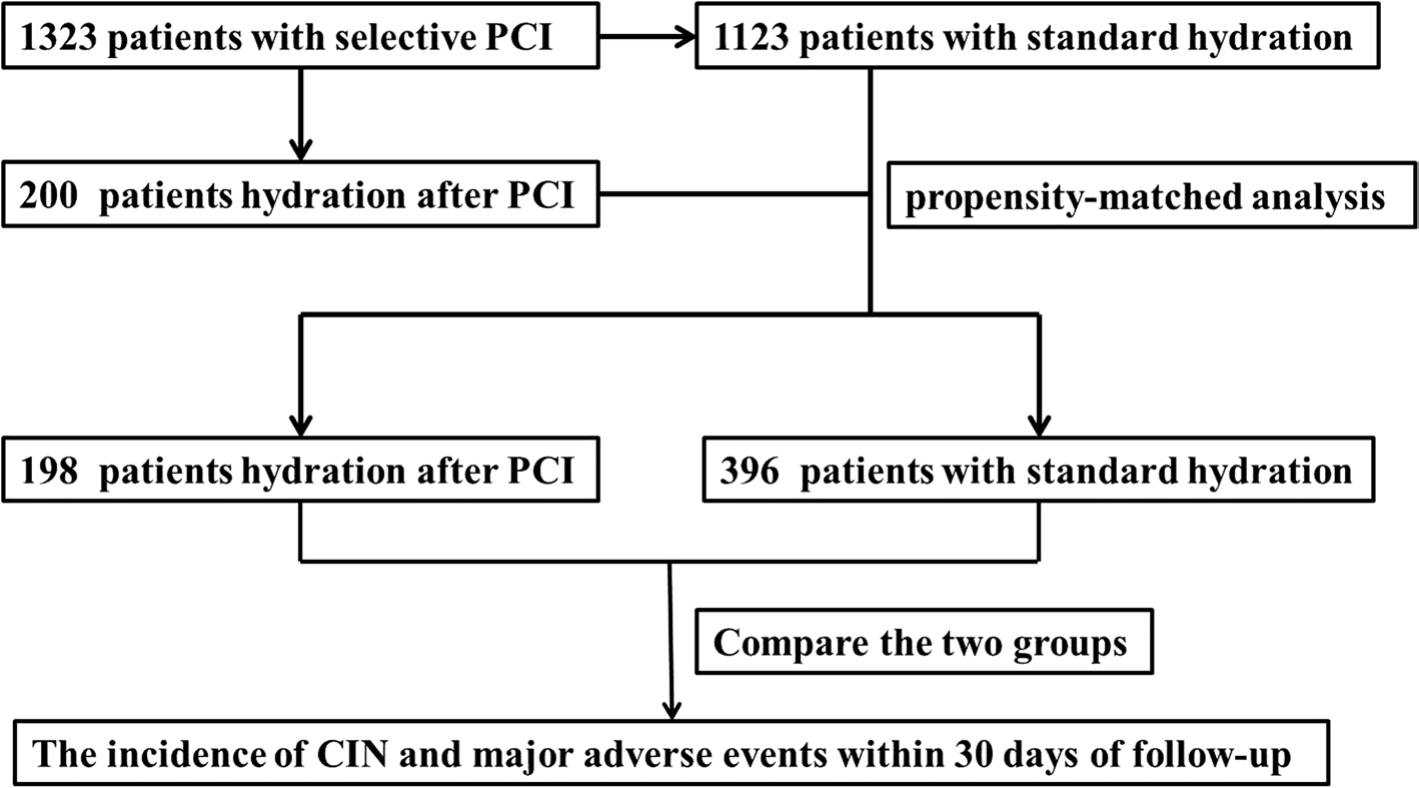
Flow diagram of the study group selection process

This study was a single-centre, retrospective, observational study. From November 2014 to November 2017, 1,323 consecutive patients from the Second Hospital of Hebei Medical University who underwent elective PCI were enrolled. There were 1,123 patients in the standard hydration group and 200 patients in the postprocedure hydration group. Using the statistical method of propensity score-matched analysis (PSM), the postprocedure hydration group was matched with the preprocedure hydration group by 1:2 according to the sex and age of the patients. There were 198 cases in the postprocedure hydration group who matched 396 cases in the preprocedure hydration group, and two cases failed (Fig. 1). The data come from a clinical study of our centre registered in the Chinese Clinical Trial Registry. The serial number was ChiCTR-IOR-14005250.

The selected patients underwent interventional therapy via the radial artery or femoral artery. Low osmolar contrast media (including iopromide, iohexol and iodofol) were used during the operation. The patients were anticoagulated with unfractionated heparin (70–100 U/kg) or bivalirudin during the procedure and treated with dual antiplatelet therapy (aspirin combined with clopidogrel or ticagrelor). All patients were treated with hydration. The preprocedure standard hydration group received an intravenous infusion of 0.9% saline at a rate of 1 or 0.5 mL/kg/h (patients with LVEF ≤ 35%) 4 h before and 20 h after elective PCI. Postprocedure hydration group: 0.9% normal saline was given immediately after PCI, and the hydration rate was the same as above, maintained until 24 h after PCI. The inclusion criteria were as follows: age 18–75 years old, regardless of sex, elective PCI, and signed informed consent. Exclusion criteria: emergency PCI, recently used contrast medium (within two days), contrast medium allergy, renal replacement therapy, acute decompensated heart failure, severe heart valve disease, after heart or kidney transplantation.

### Variables and study endpoint

The baseline clinical data and laboratory examination of 198 patients in the postprocedure hydration group and 396 patients in the standard hydration group were collected, including body mass index (BMI), left ventricular ejection fraction (LVEF), haemoglobin, glycosylated haemoglobin, low-density lipoprotein cholesterol (LDL-C), past medical history (hypertension, diabetes, hyperlipidaemia, old myocardial infarction, smoking, etc.) and combined medication (statins, angiotensin-converting enzyme inhibitors/angiotensin receptor blockers, etc.

The serum creatinine (sCr), serum urea nitrogen (BUN), estimated glomerular filtration rate (eGFR), B-type natriuretic peptide (BNP), cystatin-C, and ß2-microglobulin (ß2-MG) levels at baseline and 48 h after CM exposure were compared between the two groups. The biochemical parameters were measured by an automatic biochemical analyser (Hitachi 7600, Japan), and BNP levels were measured using commercially available BNP assay kits. The eGFR was calculated by serum creatinine (SCr) levels and the following modification: eGFR (mL/min/1.73 m^2^) = 186 × sCr^-1.154^ × age^-0.203^ (× 0.742 for females).

The incidence of CIN and major adverse events after CM exposure was compared between the two groups. CIN was defined as a 25% relative increase in SCr from baseline or an absolute increase of 44 µmol/L or 0.5 mg/dL after exposure to CM [1]. Major adverse events occurring within 30 days after CM exposure were recorded, including all-cause death, myocardial infarction, renal failure requiring dialysis, upper gastrointestinal bleeding, and stroke. All patients were followed in an outpatient clinic or contacted by telephone.

### Statistical analysis

Continuous variables were expressed as the means with standard deviation for normally distributed variables and as median with interquartile range for nonnormally distributed variables. Categorical variables are presented as percentages. Continuous variables were compared using Student’s *t* test for normally distributed values and the Mann–Whitney *U* test for nonnormally distributed values. Proportions were compared using the Chi-square test, and if the expected frequency was < 5, the Fisher exact test was applied. The variables with statistical significance in univariate analysis and those professionally considered to have an impact on the outcome were included in the multivariate logistic regression model to explore the independent influencing factors of the outcome, and the test level was 0.05. Multivariate logistic regression analysis was used to explore the independent risk factors for CIN. For the bilateral test, *P* < 0.05, the difference was considered statistically significant.

## Result

### Clinical characteristics

There were no significant differences (all *P* > 0.05) between the two groups in baseline clinical characteristics. Although the amount of CM used in the postprocedure hydration group was greater than that in the preprocedure hydration group (155.25 ± 56.66 mL vs. 147.96 ± 62.42 mL), there was no statistically significant difference (*P* = 0.563). Meanwhile, the difference in left ventricular end-diastolic pressure between the two groups was not statistically significant (*P* > 0.05) (Table 1).

**Table 1:** Baseline clinical characteristics of the patients

Variables	Hydration after PCI (*n* = 198)	Hydration before PCI (*n* = 396)	*P* value
Age(years)	58.76 ± 9.03	58.80 ± 8.96	0.959
Male, n (%)	132(66.70)	268(67.90)	0.757
BMI (kg/m^2^)	25.65 ± 3.55	25.72 ± 3.35	0.822
Hypertension, n (%)	116(58.60)	237(59.80)	0.768
Diabetes mellitus, n (%)	50(25.25)	105(26.52)	0.741
Hyperlipidaemia, n (%)	80(40.40)	177(44.70)	0.319
Smoking, n (%)	76(38.38)	136(34.34)	0.333
LVEF (%)	60.90 ± 4.94	60.95 ± 6.52	0.930
AMI, n(%)	63(31.82)	121(30.56)	0.754
Multiple vessel lesions, n(%)	105(53.03)	201(50.76)	0.601
Laboratory results			
Haemoglobin (g/L)	132.69 ± 11.09	133.93 ± 13.21	0.297
Glycosylated haemoglobin (%)	6.23 ± 1.16	6.39 ± 1.15	0.239
Low-density lipoprotein cholesterol (mg/dL)	98.89 ± 26.57	103.69 ± 33.54	0.112
Medications, n (%)			
ß-blocker	137(69.19)	250(63.13)	0.144
ACEI/ARB	108(54.54)	196(49.49)	0.246
Statins	180(90.91)	357(90.15)	0.768
Diuretics	26(13.13)	59(14.90)	0.562
Nitrate	136(68.69)	267(67.42)	0.756
Calcium-channel blocker	84(42.67)	185(46.72)	0.322
Proton pump inhibitors	53(26.77)	84(21.21)	0.130
Use of bivalirudin, n (%)	69(34.85)	121(30.56)	0.290
Use of GPI, n (%)	39(19.70)	86(21.72)	0.569
Volume of CM (mL)	155.25 ± 56.66	147.96 ± 62.42	0.167
CM = 200 mL, n (%)	38(19.19)	81(20.45)	0.717
LVEDP	16.26 ± 5.03	16.87 ± 6.37	0.205

*BMI* Body mass index, *AMI* acute myocardial infarction, *LVEF* left ventricular ejection fraction, *ACEI* angiotensin-converting enzyme inhibitors, *ARB* angiotensin receptor blockers, *PCI* percutaneous coronary intervention, *GPI* platelet glycoprotein IIb/IIIa inhibitors, *CM* contrast medium, *LVEDP* Left ventricular end-diastolic pressure

### Changes in renal function parameters and incidence of CIN

**Fig. 2:**
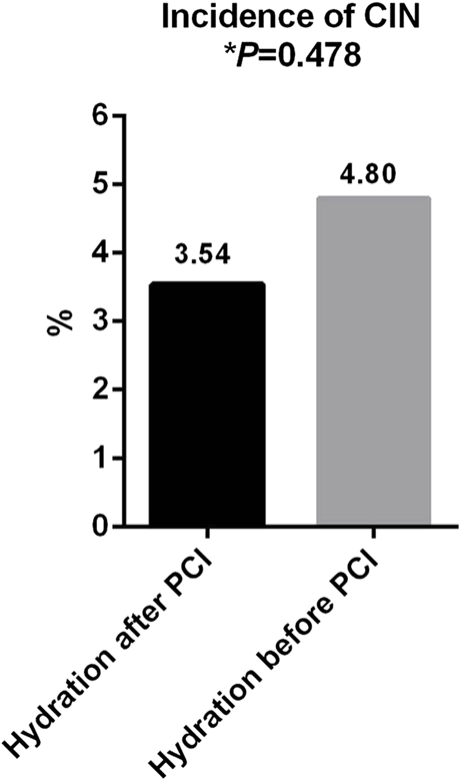
Incidence of CIN between the two groups

There was no significant difference in the baseline levels of renal function parameters (sCr, eGFR and Cys-C) between the postprocedure hydration and preprocedure hydration groups (all *P* > 0.05). The sCr and Cys-C levels were increased, and the eGFR levels were decreased at 48 h after PCI compared with baseline in both groups (all *P* < 0.05). However, there was no significant difference in sCr, eGFR and Cys-C levels at 48 h after PCI between the two groups (all *P* > 0.05). Meanwhile, there was no statistically significant difference in the incidence of CIN between the two groups (3.54% vs. 4.8%, *P* = 0.478) (Table 2, Fig. 2).

**Table 2:** Changes in sCr, eGFR and Cystatin-C, incidence of CIN

Variables	Hydration after PCI (*n* = 198)	Hydration before PCI (*n* = 396)	*P* value
sCr (µmol/L)			
Baseline	67.33 ± 14.72	69.16 ± 15.15	0.162
48 h after exposure	70.63 ± 17.59*	70.30 ± 15.37*	0.808
eGFR (mL/min/1.73m^2^)			
Baseline	105.61 ± 23.07	103.52 ± 22.55	0.223
48 h after exposure	103.08 ± 25.45*	101.29 ± 23.89*	0.542
Cystatin-C (mg/L)			
Baseline	1.03 ± 0.26	1.05 ± 0.23	0.277
48 h after exposure	1.09 ± 0.26*	1.08 ± 0.22*	0.503
Incidence of CIN, n (%)	7(3.54)	19(4.80)	0.478

*SCr* Serum creatinine, *eGFR* Estimated glomerular filtration rate, *CIN* Contrast-induced nephropathy

^*^P < 0.05 compared with baseline

### Multifactor logistic regression analysis of the predictors of CIN

Univariate analysis was performed to determine whether there were significant differences between the CIN and non-CIN groups in baseline clinical factors, including age, sex, body mass index (BMI), left ventricular ejection fraction (LVEF), B-type natriuretic peptide (BNP), blood creatinine (sCr), glomerular filtration rate (eGFR), and contrast agent dosage. The results showed that the differences between the two groups were statistically significant (*P* < 0.05) for diabetes mellitus, BNP and cystatin C levels, contrast media dosage, and BMI, while the differences between the two groups were not statistically significant (*P* > 0.05) for the remaining indicators (Table 3).

**Table 3:** Comparison of clinical characteristics between CIN and non-CIN patients

Variables	Non-CIN (*n* = 568)	CIN (*n* = 26)	t/Z/*x*^2^ value	*P* value
Hydration after PCI	189(33.3%)	7(26.9%)	0.300	0.584
Age(years)	58.85 ± 8.77	59.38 ± 8.86	-0.302	0.763
Male, n(%)	381(67.1%)	19(73.1%)	0.548	0.459
BMI	25.53 ± 3.27	27.26 ± 3.06	-2.626	0.009
Smoking, n (%)	200(35.2%)	12(46.2%)	0.655	0.418
Diabetes mellitus, n (%)	141(24.8%)	14(53.8%)	12.234	0.000
Hypertension, n (%)	339(56.7%)	14(53.8%)	0.005	0.943
Hyperlipidaemia, n (%)	246(43.3%)	11(42.3%)	0.001	0.971
AMI, n(%)	174(30.6%)	10(38.5%)	1.375	0.241
LVEF (%)	61.14 ± 5.81	59.58 ± 8.46	1.292	0.197
BNP (pg/mL)	28.80(11.50,65.70)	71.25(27.25,165.25)	-2.600	0.009
HbA1c (%)	6.02 ± 1.18	6.00 ± 0.70	0.105	0.916
Haemoglobin (g/L)	136.00 ± 12.41	133.15 ± 15.76	1.120	0.263
LDL-C(mg/dL)	102.98 ± 32.08	105.05 ± 23.31	-0.323	0.747
Cystatin-C (mg/L)	1.05 ± 0.21	1.23 ± 0.32	-3.857	0.000
sCr (µmol/L)	68.55 ± 15.49	67.40 ± 12.98	0.373	0.709
eGFR (mL/min/1.73m^2^)	104.77 ± 23.43	108.10 ± 21.48	-0.709	0.479
LVEDP(mmHg)	16.00(13.00,20.00)	15.00(10.00,18.50)	-1.332	0.183
Volume of CM (mL)	149.59 ± 62.67	198.46 ± 47.89	-3.913	0.000

*BMI* Body mass index, *AMI* Acute myocardial infarction, *BNP* B-type natriuretic peptide, *LVEF* left ventricular ejection fraction, *CM* contrast medium, *LVEDP* Left ventricular end-diastolic pressure

Meanwhile, multivariate logistic regression was used to analyse the independent risk factors associated with the incidence of CIN. The results showed that the predictors of CIN were diabetes (OR = 5.01, 95% CI 2.04–12.35, *P* < 0.001), baseline BNP (OR = 1.003, 95% CI 1.001–1.004, *P* = 0.001), baseline cystatin C (OR = 17.48, 95% CI 3.61–84.74, *P* < 0.001), and CM dosage (OR = 1.009, 95% CI 1.004–1.014, *P* = 0.001), whereas BMI was not significantly associated with the incidence of CIN (*P* = 0.197) (Table 4).

**Table 4:** Multivariate logistic regression analysis of risk factors for the development of CIN

Variables	B	S.E	Wald	P	OR	95% C.I.OR	
						lower	upper
Diabetes mellitus	1.612	0.460	12.297	0.000	5.014	2.036	12.345
Cystatin-C	0.003	0.001	11.063	0.001	1.003	1.001	1.004
BNP	2.861	0.805	12.626	0.000	17.484	3.607	84.738
Volume of CM	0.009	0.003	11.099	0.001	1.009	1.004	1.014
BMI	0.071	0.055	1.663	0.197	1.073	0.964	1.195

*BMI* Body mass index, *BNP* B-type natriuretic peptide, *CM* Contrast medium

### Clinical follow-up

The 30-day clinical follow-up revealed no significant difference in the incidence of major adverse events between the two groups (3.03% vs. 2.02%,* P* = 0.830). Five patients in the postprocedure hydration group were readmitted with acute coronary syndrome; one person developed stroke. Six people in the control group were readmitted with acute coronary syndrome events, one with myocardial infarction, and one with upper gastrointestinal bleeding. There were no deaths in either group during the follow-up period, and no patients had progressive renal insufficiency requiring dialysis.

## Discussion

Contrast-induced nephropathy (CIN) is a medically induced kidney injury that occurs 48–72 h after the application of contrast media and causes a significant increase in blood creatinine, which in severe cases may result in acute kidney injury [6]. The pathophysiological mechanisms of CIN have not been fully investigated. It is generally considered to be related to direct or indirect toxicity of the CM to the kidney tissues and its influence on haemodynamics. Contrast agents are directly toxic to renal tubular epithelial cells, causing apoptosis and necrosis. Moreover, the indirect mechanism is ischaemic-hypoxic injury caused by reduced renal perfusion, haemodynamic disorders, and release of vasoactive factor's endothelin, nitric oxide, and prostaglandins [7–9].

The kind and amount of contrast agent used for the procedure, as well as the patient's underlying illness, all influence the risk of CIN. A variety of indicators can help us anticipate and identify people who are at high risk of developing CIN so that we can take preventative actions before it happens [10, 11]. The presence of chronic kidney disease in patients prior to PCI was a significant risk factor for the incidence of CIN, with the worsening of kidney function increasing the risk of CIN [12]. A retrospective study analysing data from 985,737 patients undergoing percutaneous coronary intervention (PCI) confirmed that severe chronic kidney disease is an independent risk factor for CIN [13]. Furthermore, the rate of CIN following PCI is significantly high in patients with chronic renal disease, ranging from 10 to 20% [9], while the patients enrolled in our study with normal renal function had a low incidence of CIN of approximately 4%. Diabetes and heart failure (low ejection fraction) have been shown to be independent predictors of CIN, and patients with diabetes and/or heart failure are more likely to have chronic kidney damage [14, 15]. It was found that cystatin C is more sensitive to detect early acute kidney injury compared to serum creatinine (sCr) [16], and cystatin C generally peaks within 24 h after contrast exposure. Carlo Briguori et al. [17] showed that cystatin C aids in the early detection of CIN and assesses its prognosis. Hypotonic (iopromide, iohexol, iophorol, etc.) and isotonic (iodixanol) contrast agents reduce renal injury and can result in a significantly lower risk of CIN compared to early hyperosmotic contrast agents (pantethine) [18]. It has been shown that the occurrence of CIN is closely related to the amount of contrast agent used and that the application of excessive amounts of contrast agent (more than 350 mL or 4 mL/kg) and repeated applications of contrast agent within 72 h can significantly increase the incidence of CIN [19, 20]. This is consistent with the results of the present study, in which the contrast dosage was significantly higher in the CIN group than in the non-CIN group (198.46 ± 47. 89 mL vs. 149.59 ± 62.67 mL, *P* < 0.01), and contrast dose was one of the independent predictors of CIN. In addition, there is also evidence that the disease's clinical manifestation is linked to renal damage; for example, acute ST-segment elevation myocardial infarction (STEMI) with primaryPCI (PPCI) raises the risk of CIN [21]. CIN is associated with high mortality and morbidity and long hospital stay in patients with STEMI. And acute kidney injury (AKI) has been demonstrated in a prior study to be an independent predictor of long-term mortality in STEMI patients receiving PPCI [22]. The prediction of CIN is very important in patients with STEMI thus several parameters have been proposed to predict adverse events in these patients. Yildiz I et al. [23] found that serum osmolarity can be useful to define patients with STEMI undergoing PPCI who are more likely to develop CIN. Other studies revealed that Syntax Score II and Platelet-to-Lymphocyte Ratio were independent predictors of CIN in STEMI patients treated with PPCI [24, 25].

There are many relevant CIN prevention measures, including the application of hypotonic or isotonic contrast agents, reduction of contrast agent dosage, avoidance of coadministration of nephrotoxic drugs, and prophylactic application of antioxidant and anti-inflammatory drugs (N-acetylcysteine, statins, etc.) [26–29], while clinical volume expansion (hydration) to maintain renal perfusion is considered the cornerstone of CIN prevention [1]. Hydration strategies are currently recommended by national and international guidelines and expert consensus for CIN prevention and treatment [5]. Recent studies have shown that hydration reduces the incidence of CIN after PCI for acute ST-segment elevation myocardial infarction. Two studies enrolled 216 and 408 patients with ST-segment elevation myocardial infarction undergoing PCI, and the hydration regimen was saline 0.5–1 mL/kg/h maintained from 12 h before to 12 h after PCI. The results of the studies showed a 35% and 48% reduction in the incidence of CIN in the hydration group; moreover, a significant reduction in adverse events such as in-hospital dialysis and death were observed [30, 31]. A meta-analysis of seven clinical trials enrolling a total of 2,851 patients found that prophylactic hydration reduced the risk of CIN and all-cause mortality but not dialysis events [32]. Except for standard normal saline hydration, are there any other alternative solutions we can choose? In 2004, a small clinical trial [33] enrolled 119 patients randomized to a sodium bicarbonate hydration group and a sodium chloride hydration group to observe the incidence of CIN after contrast application, and the results showed that the incidence of CIN in the sodium bicarbonate hydration group showed a significant reduction in the incidence of CIN compared to the control group (1.7% vs. 13.6%, *P* = 0.02). The researchers predicted that sodium bicarbonate may protect the renal medulla from oxidative stress damage by scavenging oxygen-free radicals. However, numerous clinical studies, including meta-analyses, have not demonstrated the superiority of sodium bicarbonate hydration [34]. A recent large-scale clinical trial (PRESERVE) [35] enrolled 5,177 patients with renal insufficiency or diabetes mellitus undergoing contrast examination and showed no significant difference in the incidence of CIN and 90-day adverse events between the sodium bicarbonate hydration and sodium chloride hydration groups. Are there more convenient methods of hydration? A clinical study randomized 225 patients at high risk of developing CIN with coronary angiography into an isotonic sodium chloride hydration group versus an oral hydration group and found no significant difference in the incidence of CIN between the two groups (6.9% vs. 7.3%, *P* = 0.89) [36]. Another meta-analysis [37] found no significant difference in the prevention of CIN between oral and intravenous hydration, with all patients having an eGFR greater than 30 mL/min/1.73m^2^. Although oral hydration appears to give more protection than intravenous hydration, the preventative effect of CIN in individuals with chronic renal disease is unclear [38]. Our research revealed another convenient method of saline hydration, and the results showed that postprocedure hydration was as effective as standard hydration in preventing CIN (3.54% vs. 4.8%, *P* = 0.478). Meanwhile, there was no significant difference in the incidence of major adverse events between the two groups (3.03% vs. 2.02%, *P* = 0.830).

No consensus has been reached on a precise hydration strategy. The American College of Radiology guidelines recommend 100 mL/h of intravenous isotonic saline from 6 to 12 h before angiography until 4 to 12 h following angiography [4]. The European Society of Cardiology (ESC) guidelines for myocardial revascularization recommend a regimen of intravenous isotonic saline (1–1.5 mL/kg/h) from 12 h before until 12 h after the procedure [5]. According to KDIGO recommendations [39], hydration at a rate of 1 to 1.5 mL/kg/h for 3 to 12 h before contrast exposure is recommended, followed by sustained hydration for 6 to 12 h with a goal urine volume of > 150 mL/h. There is still sufficient hydration time for patients undergoing elective coronary angiography or PCI, but there is no clear agreement on how to adjust the hydration regimen for patients who do not have time to hydrate, such as those undergoing emergency PCI. Furthermore, to our knowledge, there are no clinical studies or reports on whether a single postprocedure hydration strategy is as effective as standard hydration in patients undergoing elected PCI. In this retrospective study, 198 postprocedure hydration cases were successfully matched with 396 preprocedure hydration patients using propensity-matched statistics, and the results showed that postprocedure hydration was comparable to preprocedure hydration in the prevention of CIN in patients undergoing elected PCI for coronary heart disease (3.54% vs. 4.8%, *P* = 0.478). The results of our study were similar to the oral hydration mentioned above [36, 37]. We predicted that postoperative hydration may also have an effective volume expansion, which can decrease the incidence of CIN. Our study contributes to the simplification of the hydration protocol and has important clinical significance and broad clinical application prospects.

In terms of clinical follow-up, the low incidence of serious adverse events and the absence of patients with death and progression to dialysis events in this study might be due to the following factors: all patients enrolled in this study underwent routine hydration; the recruited cohort was at low risk of CIN (baseline blood creatinine values and left cardiac function, mainly normal); and fewer patients with complex PCI were enrolled in this study.

### Limitations

There are some limitations in this study. First, our study is a retrospective study with study population limitation and many confounding factors, and further prospective randomized controlled clinical trials with large study population are needed to confirm the findings of this study. Second, most of the patients enrolled in this study were at low risk of CIN and had normal baseline renal function, and there were few patients with severe renal insufficiency. Further enrolments of patients with combined chronic renal insufficiency and elevated baseline creatinine are needed to clarify the preventive effect of postprocedure hydration in the high-risk group of CIN. Again, the use of hypotonic contrast agents in this investigation, with no restrictions on the kind or brand of contrast agents, may have influenced the findings.

## Conclusion

In conclusion, we demonstrated that postprocedure hydration was as effective as standard hydration in the prevention of CIN in patients with coronary heart disease undergoing elective PCI. These patients may have potential benefits from the simplified hydration method.

## Supplementary Information

**Additional file 1.**

## Data Availability

Data relevant to this study are available from the corresponding authors upon reasonable request.

## References

[1] Mehran R, Dangas GD, Weisbord SD (2019). Contrast-Associated Acute Kidney Injury. N Engl J Med.

[2] Cai Q, Jing R, Zhang W, Tang Y, Li X, Liu T (2020). Hydration Strategies for Preventing Contrast-Induced Acute Kidney Injury: A Systematic Review and Bayesian Network Meta-Analysis. J Interv Cardiol.

[3] Chandiramani R, Cao D, Nicolas J, Mehran R (2020). Contrast-induced acute kidney injury. Cardiovasc Interv Ther.

[4] Kodzwa R (2019). ACR Manual on Contrast Media: 2018 Updates. Radiol Technol.

[5] Windecker S, Kolh P, Alfonso F, Collet JP, Cremer J, Falk V (2015). 2014 ESC/EACTS guidelines on myocardial revascularization. EuroIntervention.

[6] Rear R, Bell RM, Hausenloy DJ (2016). Contrast-induced nephropathy following angiography and cardiac interventions. Heart.

[7] Mamoulakis C, Tsarouhas K, Fragkiadoulaki I, Heretis I, Wilks MF, Spandidos DA, et al**.** Contrast-induced nephropathy: Basic concepts, pathophysiological implications and prevention strategies. Pharmacology and Therapeutics. 2017:S0163725817301572. 10.1016/j.pharmthera.2017.06.009 28642116

[8] Briguori C, Donnarumma E, Quintavalle C, Fiore D, Condorelli G (2015). Contrast-induced acute kidney injury: potential new strategies. Curr Opin Nephrol Hypertens.

[9] Azzalini L, Spagnoli V, Ly HQ (2015). Contrast-Induced Nephropathy: From Pathophysiology to Preventive Strategies. Can J Cardiol.

[10] Satilmis S, Karabulut A (2020). Value of C-Reactive Protein/Albumin Ratio in Predicting the Development of Contrast-Induced Nephropathy in Patients With Non-ST Elevation Myocardial Infarction. Angiology.

[11] Wei W, Zhang L, Zhang Y, Tang R, Zhao M, Huang Z (2021). Predictive value of creatine kinase MB for contrast-induced acute kidney injury among myocardial infarction patients. BMC Cardiovasc Disord.

[12] McCullough PA, Adam A, Becker CR, Davidson C, Lameire N, Stacul F (2006). Epidemiology and prognostic implications of contrast-induced nephropathy. Am J Cardiol.

[13] Tsai TT, Patel UD, Chang TI, Kennedy KF, Masoudi FA, Matheny ME (2014). Contemporary incidence, predictors, and outcomes of acute kidney injury in patients undergoing percutaneous coronary interventions: insights from the NCDR Cath-PCI registry. JACC Cardiovasc Interv.

[14] Silver SA, Shah PM, Chertow GM, Harel S, Wald R, Harel Z (2015). Risk prediction models for contrast induced nephropathy: systematic review. BMJ.

[15] Han Y, Zhu G, Han L, Hou F, Huang W, Liu H (2014). Short-term rosuvastatin therapy for prevention of contrast-induced acute kidney injury in patients with diabetes and chronic kidney disease. J Am Coll Cardiol.

[16] Fu N, Liang M, Yang S (2018). High Loading Dose of Atorvastatin for the Prevention of Serum Creatinine and Cystatin C-Based Contrast-Induced Nephropathy Following Percutaneous Coronary Intervention. Angiology.

[17] Briguori C, Visconti G, Rivera NV, Focaccio A, Golia B, Giannone R (2010). Cystatin C and contrast-induced acute kidney injury. Circulation.

[18] Faucon AL, Bobrie G, Clément O (2019). Nephrotoxicity of iodinated contrast media: From pathophysiology to prevention strategies. Eur J Radiol.

[19] Maioli M, Toso A, Gallopin M, Leoncini M, Tedeschi D, Micheletti C (2010). Preprocedural score for risk of contrast-induced nephropathy in elective coronary angiography and intervention. J Cardiovasc Med (Hagerstown).

[20] Mehran R, Aymong ED, Nikolsky E, Lasic Z, Iakovou I, Fahy M (2004). A simple risk score for prediction of contrast-induced nephropathy after percutaneous coronary intervention: development and initial validation. J Am Coll Cardiol.

[21] Sgura FA, Bertelli L, Monopoli D, Leuzzi C, Guerri E, Spartà I (2010). Mehran contrast-induced nephropathy risk score predicts short- and long-term clinical outcomes in patients with ST-elevation-myocardial infarction. Circ Cardiovasc Interv.

[22] Hayıroğlu Mİ, Bozbeyoglu E, Yıldırımtürk Ö, Tekkeşin Aİ, Pehlivanoğlu S (2020). Effect of acute kidney injury on long-term mortality in patients with ST-segment elevation myocardial infarction complicated by cardiogenic shock who underwent primary percutaneous coronary intervention in a high-volume tertiary center. Turk Kardiyol Dern Ars.

[23] Yildiz I, Yildiz PO, Rencuzogullari I, Karabag Y, Cagdas M, Burak C (2019). Association of serum osmolarity with contrast-induced nephropathy in patients with ST-segment elevation myocardial infarction. Angiology.

[24] Rencuzogullari I, Çağdaş M, Karakoyun S, Karabağ Y, Yesin M, Gürsoy MO (2018). Association of Syntax Score II with contrast-induced nephropathy and hemodialysis requirement in patients with ST segment elevation myocardial infarction undergoing primary percutaneous coronary intervention. Korean Circ J.

[25] Velibey Y, Oz A, Tanik O, Guvenc TS, Kalenderoglu K, Gumusdag A (2017). Platelet-to-Lymphocyte Ratio Predicts Contrast-Induced Acute Kidney Injury in Patients With ST-Segment Elevation Myocardial Infarction Undergoing Primary Percutaneous Coronary Intervention. Angiology.

[26] Pranata R, Tondas AE, Vania R, Toruan MPL, Lukito AA, Siswanto BB (2020). Remote ischemic preconditioning reduces the incidence of contrast-induced nephropathy in patients undergoing coronary angiography/intervention: Systematic review and meta-analysis of randomized controlled trials. Catheter Cardiovasc Interv.

[27] Zhang F, Lu Z, Wang F (2020). Advances in the pathogenesis and prevention of contrast-induced nephropathy. Life Sci.

[28] Zeng Z, Zhang H, Zhang P, Li Y, Fu N (2021). Standard vs. double dose of intravenous nicorandil in preventing contrast-induced nephropathy in patients with coronary heart disease undergoing elective coronary procedures. Coron Artery Dis.

[29] Walker H, Guthrie GD, Lambourg E, Traill P, Zealley I, Plumb A (2022). Systematic review and meta-analysis of prophylaxis use with intravenous contrast exposure to prevent contrast-induced nephropathy. Eur J Radiol.

[30] Luo Y, Wang X, Ye Z, Lai Y, Yao Y, Li J (2014). Remedial hydration reduces the incidence of contrast-induced nephropathy and short-term adverse events in patients with ST-segment elevation myocardial infarction: a single-center, randomized trial. Intern Med.

[31] Jurado-Román A, Hernández-Hernández F, García-Tejada J, Granda-Nistal C, Molina J, Velázquez M (2015). Role of hydration in contrast-induced nephropathy in patients who underwent primary percutaneous coronary intervention. Am J Cardiol.

[32] Jiang Y, Chen M, Zhang Y, Zhang N, Yang H, Yao J (2017). Meta-analysis of prophylactic hydration versus no hydration on contrast-induced acute kidney injury. Coron Artery Dis.

[33] Merten GJ, Burgess WP, Gray LV, Holleman JH, Roush TS, Kowalchuk GJ (2004). Prevention of contrast-induced nephropathy with sodium bicarbonate: a randomized controlled trial. JAMA.

[34] Zhang B, Liang L, Chen W, Liang C, Zhang S (2015). The efficacy of sodium bicarbonate in preventing contrast-induced nephropathy in patients with pre-existing renal insufficiency: a meta-analysis. BMJ Open.

[35] Weisbord SD, Gallagher M, Jneid H, Garcia S, Cass A, Thwin SS (2018). Outcomes after Angiography with Sodium Bicarbonate and Acetylcysteine. N Engl J Med.

[36] Akyuz S, Karaca M, Kemaloglu Oz T, Altay S, Gungor B, Yaylak B (2014). Efficacy of oral hydration in the prevention of contrast-induced acute kidney injury in patients undergoing coronary angiography or intervention. Nephron Clin Pract.

[37] Zhang W, Zhang J, Yang B, Wu K, Lin H, Wang Y (2018). Effectiveness of oral hydration in preventing contrast-induced acute kidney injury in patients undergoing coronary angiography or intervention: a pairwise and network meta-analysis. Coron Artery Dis.

[38] Novak JE, Handa R (2019). Contrast Nephropathy Associated with Percutaneous Coronary Angiography and Intervention. Cardiol Clin.

[39] Lameire N, Kellum JA (2013). Contrast-induced acute kidney injury and renal support for acute kidney injury: a KDIGO summary (Part 2). Crit Care.

